# Pediatric orbital fractures in Singapore: demographics, etiology, and the role of bioresorbable implants

**DOI:** 10.3389/fopht.2025.1506445

**Published:** 2025-03-13

**Authors:** Emmanuel Lee Boniao, Alexander Gungab, Blanche Xiao Hong Lim, Gangadhara Sundar

**Affiliations:** ^1^ Orbit & Oculofacial Surgery, Department of Ophthalmology, National University Hospital, National University of Singapore, Singapore, Singapore; ^2^ Department of Ophthalmology, Amai Pakpak Medical Center, Marawi, Philippines; ^3^ Department of Ophthalmology, Northern Mindanao Medical Center, Cagayan de Oro, Philippines; ^4^ Department of Ophthalmology, Western Visayas Medical Center, Iloilo, Philippines; ^5^ Department of Ophthalmology, Fatima University Medical Center, Antipolo, Philippines

**Keywords:** orbital fractures, orbitofacial fractures, pediatric orbital fractures, white-eye blow out fractures, entrapment, orbital implants, bioresorbable implants, orbital trauma orbital fractures

## Abstract

**Purpose:**

This study aimed to analyze the demographic characteristics, etiology, fracture types, interventions and treatment outcomes, with a focus on the increasing role of bioresorbable implants compared to traditional non-resorbable implants in pediatric orbital fractures.

**Methods:**

This was a retrospective cohort study conducted at the Department of Ophthalmology, National University Hospital, Singapore, of pediatric patients (aged 18 years or younger) treated surgically for orbital fractures from January 2005 to May 2023. Data was extracted from the hospital’s electronic medical records, including demographic details, causes of fractures, types of fractures, implants used (bioresorbable and non-bioresorbable), and clinical outcomes.

**Results:**

43 cases of pediatric orbital and orbitofacial fractures met the criteria undergoing surgical intervention. Most occurred in males (81.4%, n=35). Causes of fractures were sports-related incidents and play (46.5%, n=20), assault (30.2%, n=13), road-traffic accidents (16.3%, n=7), and non-play related accidents (7%, n=3). Most pediatric orbital fractures were unilateral (88%, n=38). While most were pure or simple orbital fractures (74.4% n=32), 25.6% (n=11) were complex orbitofacial fractures. Amongst simple orbital fractures, blowout fractures (91%, n=29) were the most common, involving the inferior (58.6%, n=17), combined floor and medial wall (20.6%, n=6), medial wall (13.8%, n=4) and roof (6.9%, n=2). Amongst the complex fractures, zygomaticomaxillary complex fractures were the most frequent (45.4%, n=5), followed by cranioorbital fractures (27.3%, n=3) and Le Fort II & III fractures (27.3%, n=3). Orbital tissue entrapment was common (56%, n=24), and most patients with entrapment underwent urgent surgical intervention (65%, n=28), usually within 24 hours (53%, n=23). The majority of those who underwent surgery had implants placed (89%, n=25), with most being bioresorbable (64.3%, n=18). All patients (100%) who underwent surgery showed clinical improvement without significant complications.

**Conclusion:**

Although simple pediatric orbital blowout fractures are still the most common among pediatric patients, the study showed that a quarter of them presented with complex orbitofacial fractures requiring multidisciplinary management. Most fractures occurred in males and typically associated with increasing play and physical activity in teenagers. The study also showed that early intervention is crucial to better outcome, with the increasing role of bioresorbable implants in this population reducing long term implant related complications.

## 1 Introduction

The adage that children are not miniature adults is particularly relevant in the context of pediatric orbital fractures (1). In early childhood, fractures of the orbital roof are more frequent compared to older children and adults, due to their larger skulls and the poorly developed frontal sinuses. As children mature and the maxillae grow, proportional differences between the skull and face decrease, and fractures to the lower part of the orbit become more common (1).

In adults, orbital fractures are often accompanied by significant periorbital edema with ecchymosis, subconjunctival hemorrhage, with or without diplopia. However, children with these types of fractures may experience diplopia and limited ocular motility, with little to no periorbital edema or visible signs of injury to the eye itself being referred to as a ‘white-eyed’ blowout fracture (2). Orbital tissues including orbital fat, extraocular muscle and intermuscular septum (EOM-IMS Complex) may be incarcerated resulting to diplopia (2), and is often associated with nausea, vomiting, and bradycardia, which are signs of the oculocardiac reflex (3).

Although pediatric orbital fractures are present in 15% of pediatric fractures in general (4), the mechanisms of the full spectrum of pediatric orbital fractures, not just orbital floor blowout fractures, are rarely reported in literature. Previous studies emphasize the vulnerability of pediatric orbit to fractures from low-energy impacts (4). Typical causes include sports-related activities, falls, and motor vehicle accidents. High-impact sports, particularly those involving balls or physical assaults (5), falls from playground equipment or bicycles (6) and motor vehicle accidents (7) are various causes of orbital fractures. Losee et al. in a North American study found that activities of daily living (36.5%) were the most common cause, followed by motor vehicle accidents (29.7%), sports (24.3%), and violence (9.5%) (8). However, data in various Asian populations is limited.

Apart from the commonly mentioned orbital floor blowout fractures in children, there is no universally accepted method for classifying fractures of the orbital walls and their complexity. This lack of classification has resulted in a grouping of orbital fractures with either complex facial fractures by plastic surgeons or isolated blowout fractures by ophthalmologists, without a middle ground for both. Herewith we have described and use the Practical classification of orbital and orbitofacial fractures (9) broadly classifying fractures into simple or pure orbital wall fractures and complex orbitofacial fractures involving the rim and adjacent craniofacial skeleton. Simple fractures include linear fractures, one or two wall blow-out fractures and blow-in fractures, with intact orbital rims. Complex fractures on the other hand are fractures that involve the orbital rims along with 2 or more orbital walls with involvement of the adjacent facial skeleton (9). Examples of complex orbitofacial fractures include zygomatico-maxillary complex (ZMC) fractures, naso-orbito-ethmoidal (NOE) fractures, Le Fort type fractures (type II & III), cranioorbital fractures and panfacial fractures. This is particularly important as simple fractures can be managed by one specialty (e.g. Oculoplastic Surgeons), but complex fractures may require multidisciplinary intervention (9).

Orbital fractures, especially those with tissue or muscle incarceration, should be repaired as early as possible for the best prognosis (10). The choice of surgical intervention and materials used in the repair of pediatric orbital fractures has evolved. Traditional non-resorbable implants, while effective and provide robust support, carry risks of palpability, infection, migration, extrusion, and interference with facial growth (6, 11). The advent of bioresorbable implants offers a promising alternative, facilitate natural bone healing, with timely resorption avoiding long term complications and need for subsequent surgeries (12). Studies have shown that synthetic bioresorbable implants are just as safe and effective as permanent implants in adult populations with neo-osteogenesis (13). However, their role and efficacy in pediatric orbital fracture management require more extensive exploration and documentation to establish them as a standard of care (14).

The primary objective of this retrospective study is to to describe the demographics of the pediatric orbital fractures in Singapore, its etiology, classification profile and clinical outcomes, with a special focus on the role of bioresorbable implants. By providing insights into the epidemiological trends and treatment approaches for pediatric orbital fractures, this study aims to enhance clinical decision-making, optimize surgical strategies, and contribute to the growing body of evidence supporting the use of bioresorbable implants. The study findings may help guide future treatment protocols, improve patient outcomes, and inform healthcare policies for managing pediatric orbital fractures in the region and beyond.

## 2 Methods

This study was approved by the Institutional Review Board and followed the tenets of Declaration of Helsinki. This retrospective cohort study was conducted to investigate pediatric orbital fractures seen at the National University Hospital, Singapore, from January 2005 to May 2023. The study was designed to analyze various aspects of orbital fractures, including demographic data, causative factors, types of fractures, treatment methods employed, and clinical outcomes, with a specific focus on the utilization and effectiveness of bioresorbable implants versus traditional non-resorbable implants.

Inclusion criteria encompassed patients aged 18 years or younger at the time of injury, with a radiologically confirmed diagnosis of orbital fracture based on CT imaging of the orbits and face. The imaging protocol for all patients was non-contrast CT scan of the face and orbits with Image Guidance Surgery (IGS) protocol with coronal, axial and sagittal soft tissue and bone windows. All study patients exhibited clinically significant symptoms such as diplopia, restricted eye movement and orbital wall defects of variable sizes with or without radiologic signs of extraocular muscle-orbital content entrapment, all of whom underwent surgical intervention. Visual acuity was not used as a criterion. Patients who had irretrievable medical records, less than 6 months postoperative follow up, and those with preexisting craniofacial abnormalities were excluded from the study. Classification of fractures studied were based on the Practical Classification of Orbital and Orbitofacial fractures (9). These were broadly classified as Simple or Pure orbital fractures, and complex orbitofacial fractures which includes Zygomaticofacial fractures, LeFort Fractures, and cranio-orbital fractures. All surgeries were performed under general anesthesia. Pure orbital fractures were repaired through an inferior transconjunctival approach (orbital floor and combined orbital floor-medial wall fractures) or upper eyelid crease approach (orbital roof fractures). Complex orbitofacial fractures underwent both a transconjunctival, transpalpebral and transbuccal approach.

The primary sources of data for this retrospective study were electronic medical records - Clinical Patient Support System (CPSS^®^) and the Electronic Patient Information Center (EPIC^®^), housing medical and surgical details pertinent to patient care. Medical record information retrieved and analyzed included patient demographic information (age, gender, ethnicity), fracture (type, location, cause of injury, degree of displacement, tissue entrapment), surgical treatment details (type of surgery, approach, nature of implant used, timing of the surgery), and post-operative outcomes (vision, motility, diplopia resolution, second interventions, recovery status and complications).

Data collected was anonymized and coded to maintain patient confidentiality before analysis which included a combination of descriptive and inferential statistical methods to assess the relationships between demographic factors, causes of fractures, types of fractures, surgical interventions, and clinical outcomes. These include Chi-square test for laterality, outcomes and relationship between age and fracture types, T-test for age comparisons, and ANOVA for comparison between different types of etiology. The analysis was conducted using the Statistical Package for the Social Sciences (SPSS), version 26.

## 3 Results

### 3.1 Descriptive statistics

#### 3.1.1 Demographic characteristics

A total of 43 pediatric patients who underwent surgical treatment for orbital and orbitofacial fractures at the Orbit & Oculofacial Service, Dept of Ophthalmology, National University Hospital, Singapore, between January 2005 and May 2023 were included in the study. The demographic breakdown of these patients revealed distinct patterns:

#### 3.1.2 Age distribution

**Figure 1:**
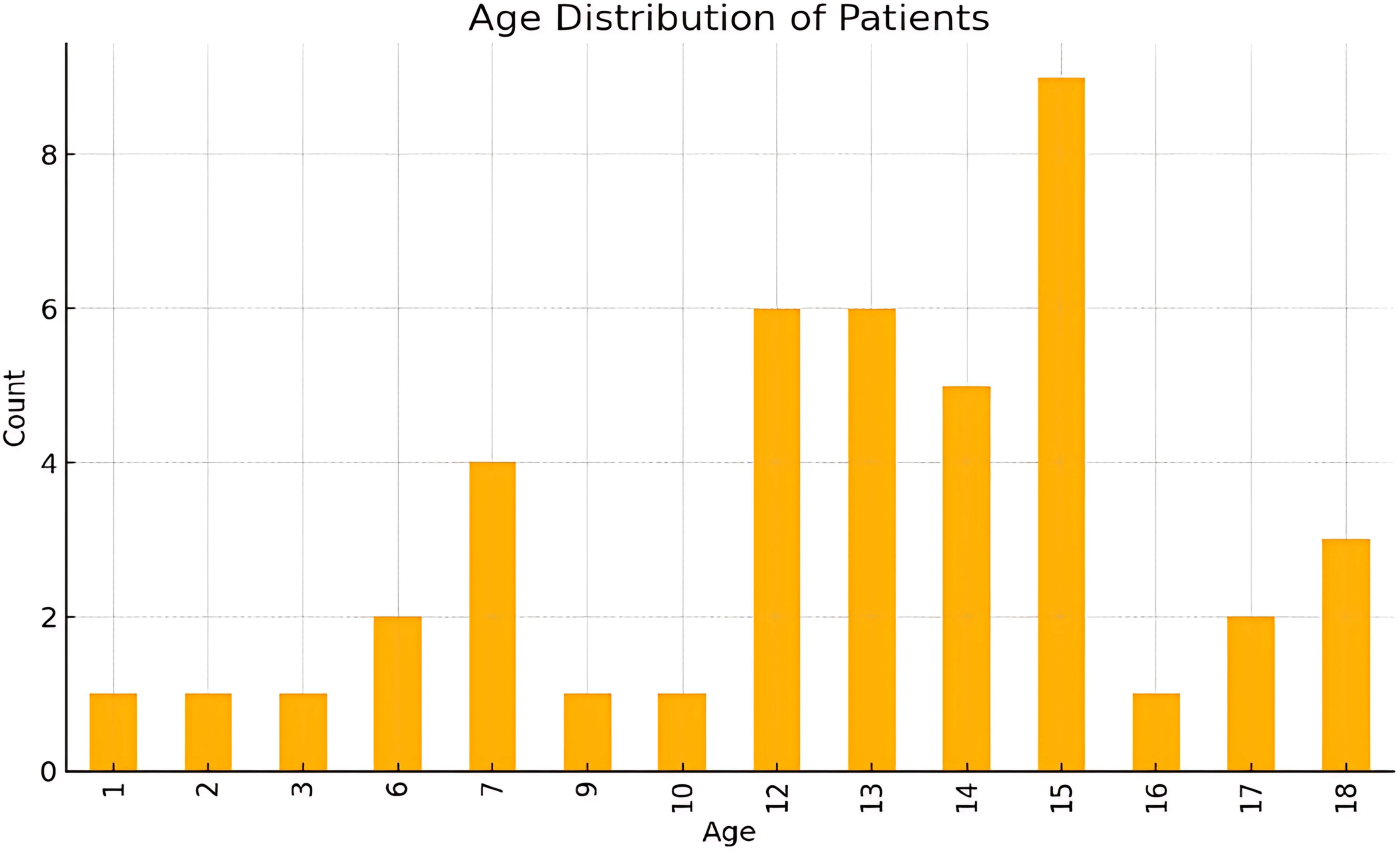
Age distribution.

The dataset included 43 patients with an average age of 12.19 years (SD 4.27, indicating variability around the mean) (**Figure 1**). Age range was 1 to 18 years, with the 25th percentile at 11 years, the 75th percentile at 15 years and a median of 13 years,. The majority of patients fell within the 12-18 year age group (74.4%), indicating a higher prevalence of orbital fractures among older children and adolescents.

Most fractures occur in early to mid-adolescence, with ages 12-15 showing the highest frequencies. Younger ages (1-3 yrs) and older teens (16-18 yrs) had relatively fewer cases. Ages 6 -10 had moderate counts, representing a middle ground between the younger and peak adolescent years.

Young children (1-6 years) of the pre-school ae group comprised 11.6% of the cases (n=5), with this group typically experiencing injuries from falls or low-energy impacts at home or in playground settings.

Pre-adolescents (7-12 years) comprised 27.9% of the cases (n=12). Injuries in this age group were often related to sports and recreational activities, as well as accidents involving bicycles and other forms of physical play. This age group coincides with primary school education in Singapore.

Adolescents (13-18 years) comprised the largest group, accounting for 60.5% of the cases (n=26). Adolescents were most frequently injured during sports, particularly contact sports, or from motor vehicle accidents. This age group coincides with secondary school education and higher levels of education in Singapore.

#### 3.1.3 Gender and ethnicity distribution 

**Figure 2:**
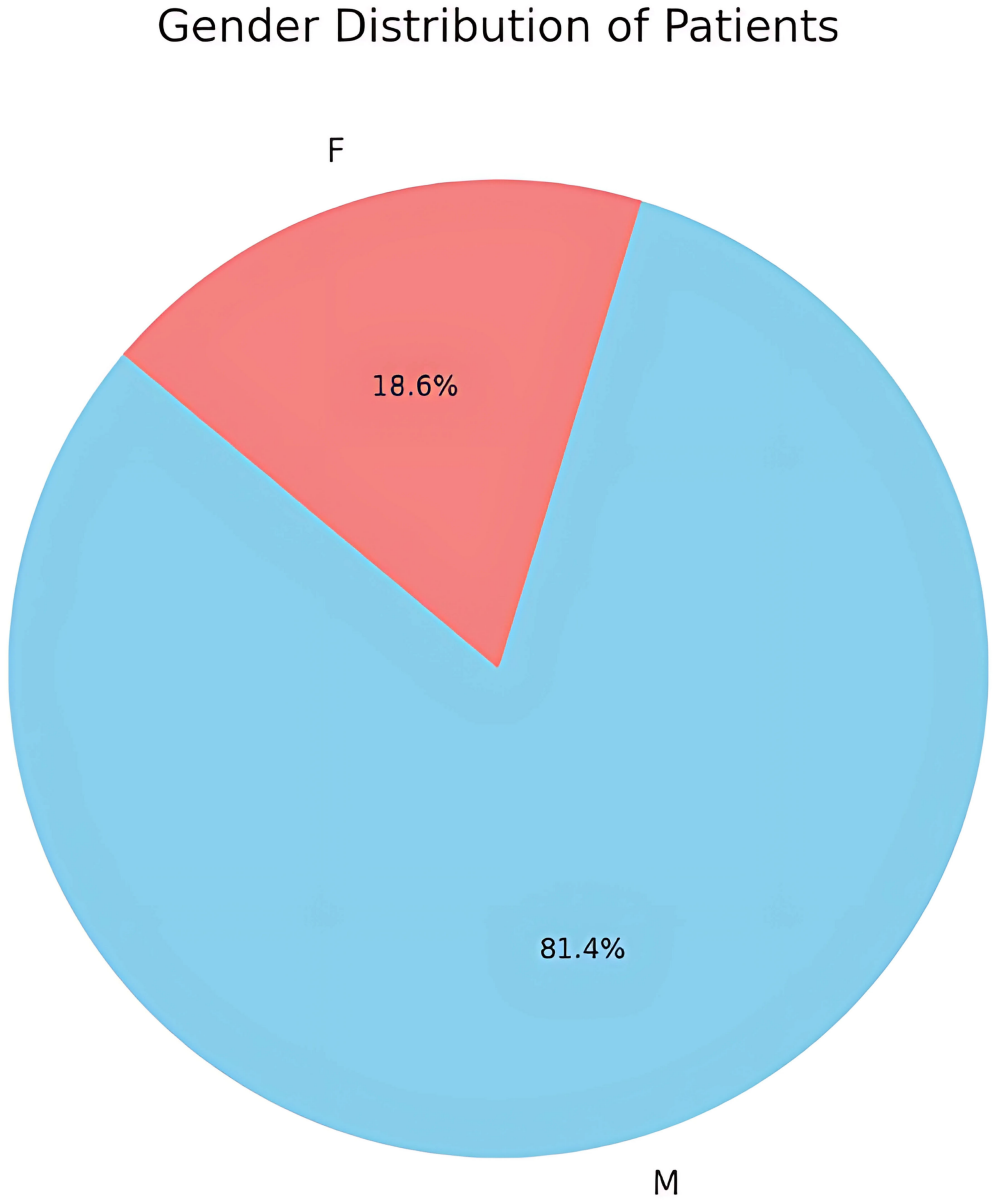
Gender distribution.

The study revealed a marked gender disparity in the incidence of pediatric orbital fractures, with males comprising 81.4% (35 out of 43) of the cases (**Figure 2**). This pronounced prevalence suggests that boys are more frequently involved in physical and high-risk activities predisposing to orbital fractures. Females comprised only 18.6% (n=8) of orbital fractures primarily from falls and sports activities.

**Figure 3:**
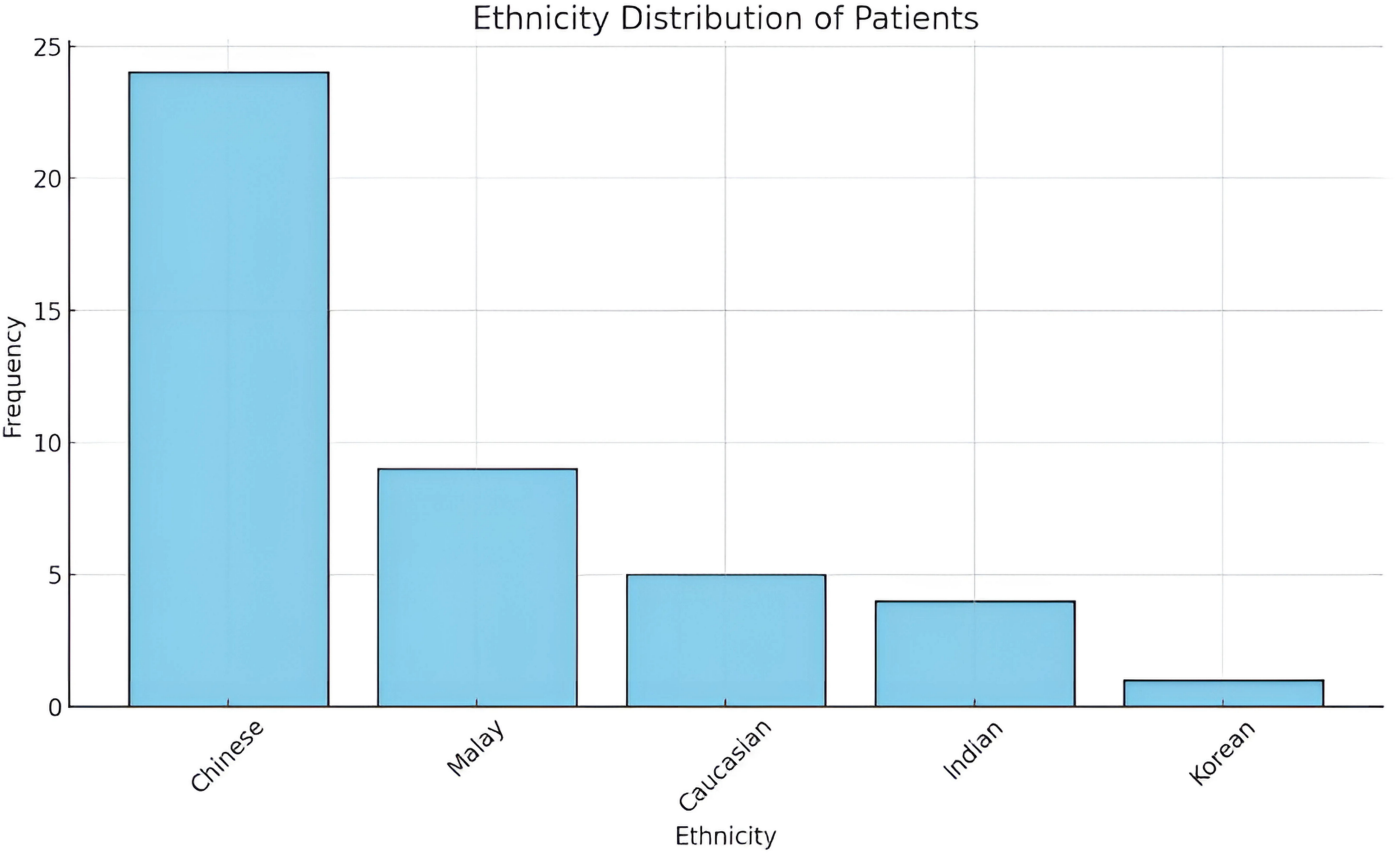
Ethnicity distribution.

Majority of patients were of Chinese descent (n=24), followed by Malays (n=9), Caucasians (n=5), Indians (n=4), and Korean (n=1) (**Figure 3**). This distribution mirrors the general demographic composition of Singapore, indicating that orbital fractures occur across all major ethnic groups but are proportionate to their population representation with the minor exception of Caucasian children slightly higher and disproportionate to ethnic distribution of the country.

#### 3.1.4 Causes of orbital fractures 

**Figure 4:**
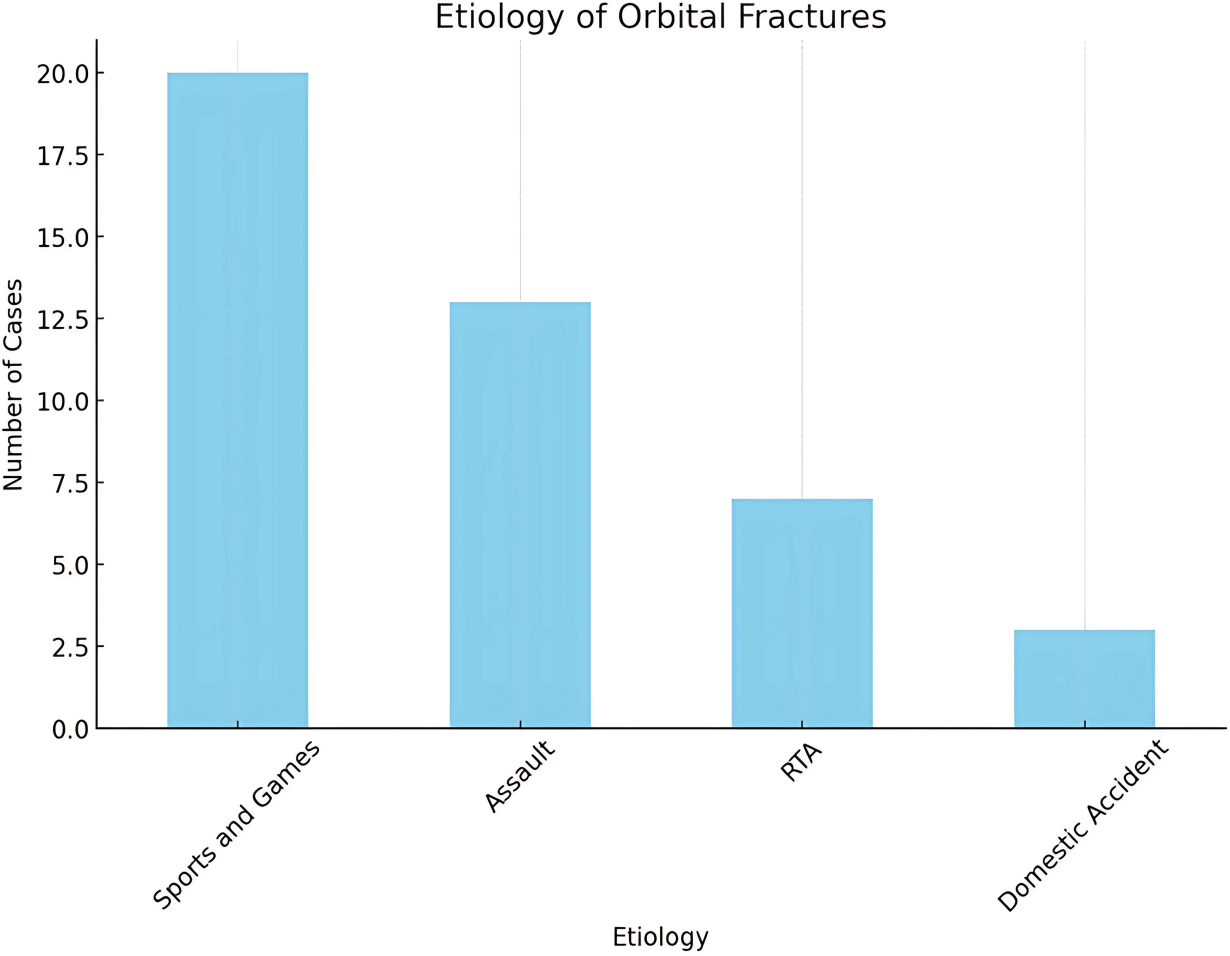
Etiology of orbital fractures.

Sports-related accidents and games were the most common cause, accounting for 46.5% (n=20) of all fractures. Commonly encountered sports included soft-ball, soccer, basketball, rugby, skiing, as well as normal play like jumping, not all of them typically considered as contact sports. Most injuries were result of normal to rough play with 70% (n=14) resulting in blowout fractures (**Figure 4**).

Assaults accounted for the next largest cause of orbital fractures - 30.2% (n=13). These were more frequent in adolescent males and occurred most commonly in school settings or as a result of interpersonal violence during play. Examples of these included punching incidents from playmates or schoolmates, and riots. Interestingly, 85% (n=11) of assaults were seen in males all of which resulted in blowout fracture.

Road traffic accidents (RTA) accounted for 16.3% (n=7) of the cases and were noted across all age groups. However, they were more prevalent among adolescents who engaged in cycling or were passengers in motor vehicles. 71% (n=5) of these patients presented with complex orbitofacial fractures such as ZMC, Le Fort, and cranio-orbital fractures.

Domestic accidents accounted for 7% (n=3) of the cases. These includes fall and fainting related to accidents.

#### 3.1.5 Fracture classification

**Figure 5:**
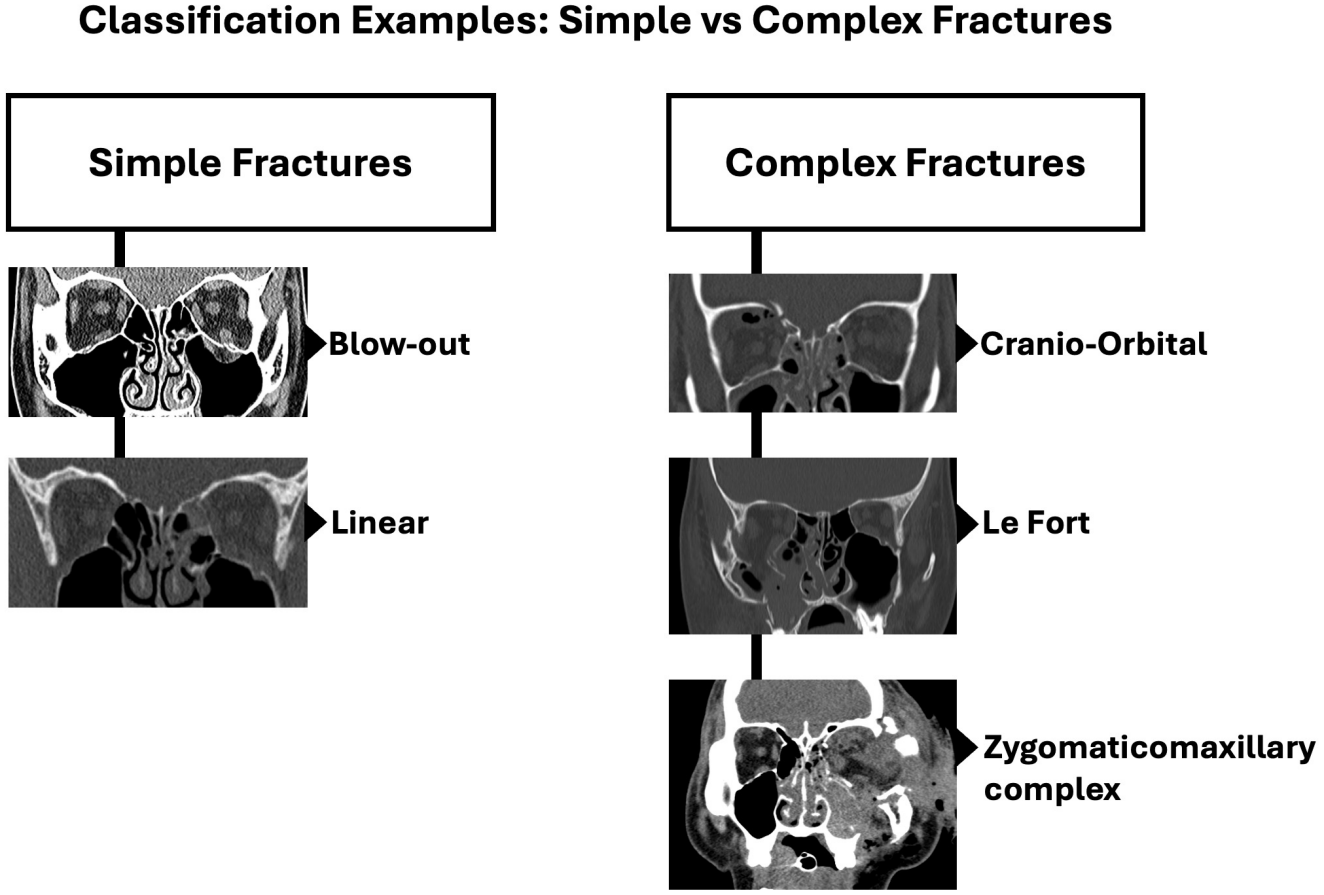
Examples of simple or pure orbital fractures vs complex orbitofacial fractures.

Classification of fractures studied were based on the Practical Classification of Orbital and Orbitofacial fractures (9). Broadly classified as simple or pure orbital fractures and complex orbitofacial fractures. Examples of simple and complex fractures are shown in **Figure 5**.

**Figure 6:**
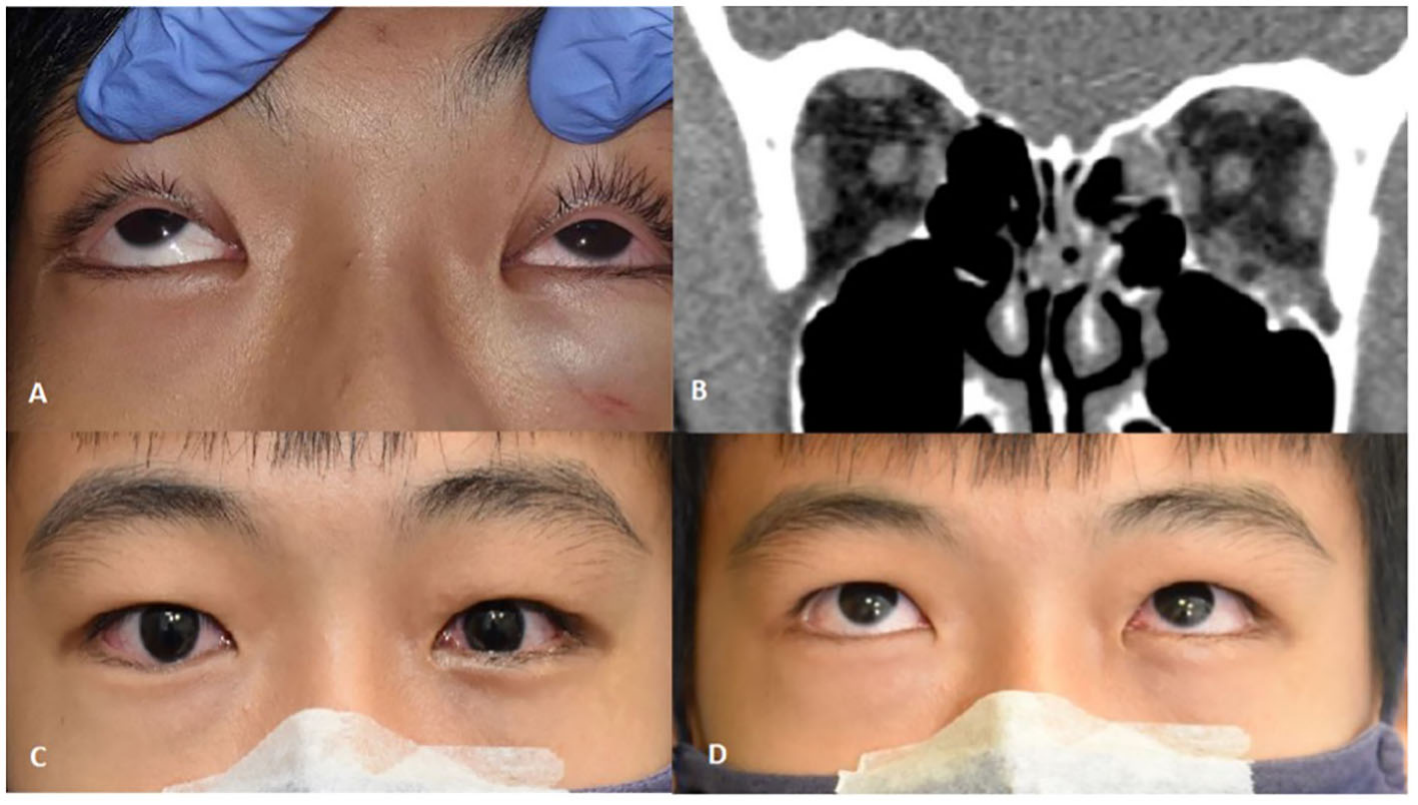
**(A)** Upgaze limitation on the left side due to tissue entrapment with symptomatic diplopia. **(B)** Coronal CT-scan of orbits soft tissue window showing left medial wall and orbital floor fracture with rounding of inferior rectus and soft tissue swelling at the left orbital floor. **(C, D)** 1 week post-transconjunctival repair with bioresorbable implant showing minimal swelling and improvement in motility.

The majority of pediatric orbital fractures seen were unilateral, representing 88% (n=38) of cases. Simple orbital fractures accounted for 74.4% (n=32) of all cases seen. These were encountered more commonly following assaults (85%) and sport-related injuries (70%). Of these, 91% (n=29) were blowout fractures, most commonly involving the inferior wall (orbital floor or roof of maxillary sinus) at 58.6%, combined floor and medial wall fractures at 20.6% (n=6) (**Figure 6**), isolated medial wall fractures at 13.8% (n=4) and the orbital roof fractures at 6.9% (n=2). Linear crack fractures occurred in 9.3% (n=3) only. No blow-in fractures were encountered in this study.

**Figure 7:**
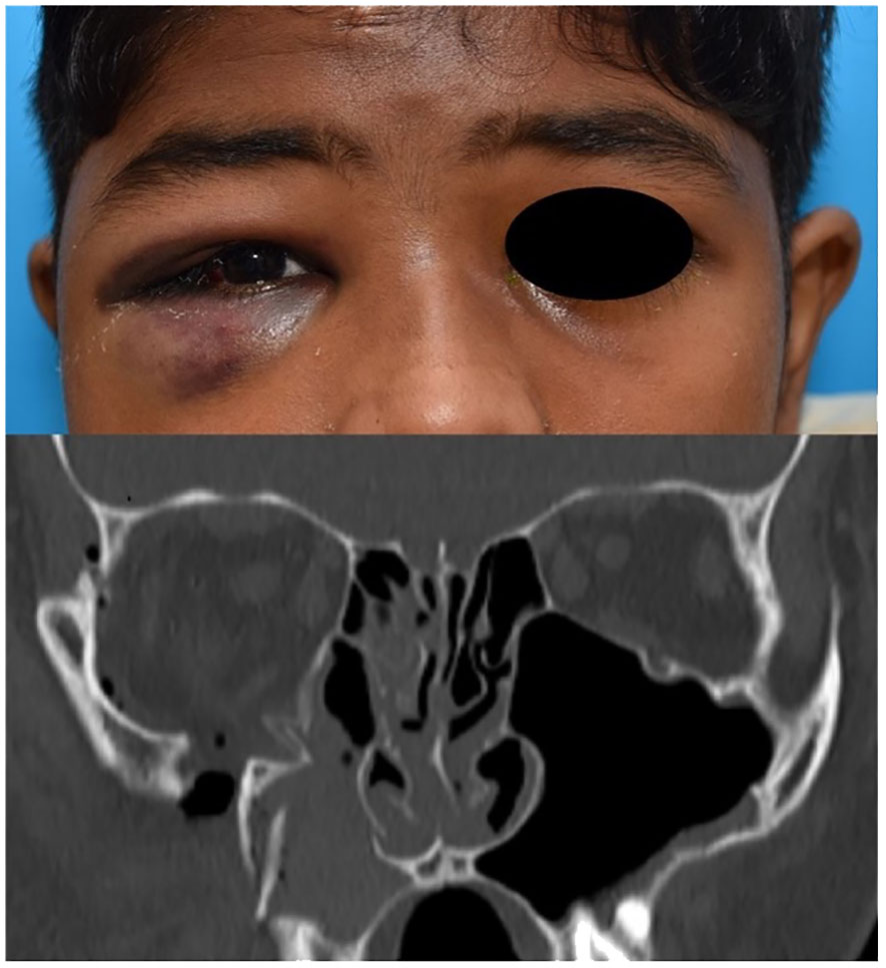
ZMC fracture of the right side with coronal bone window image showing displacement with comminution.

**Figure 8:**
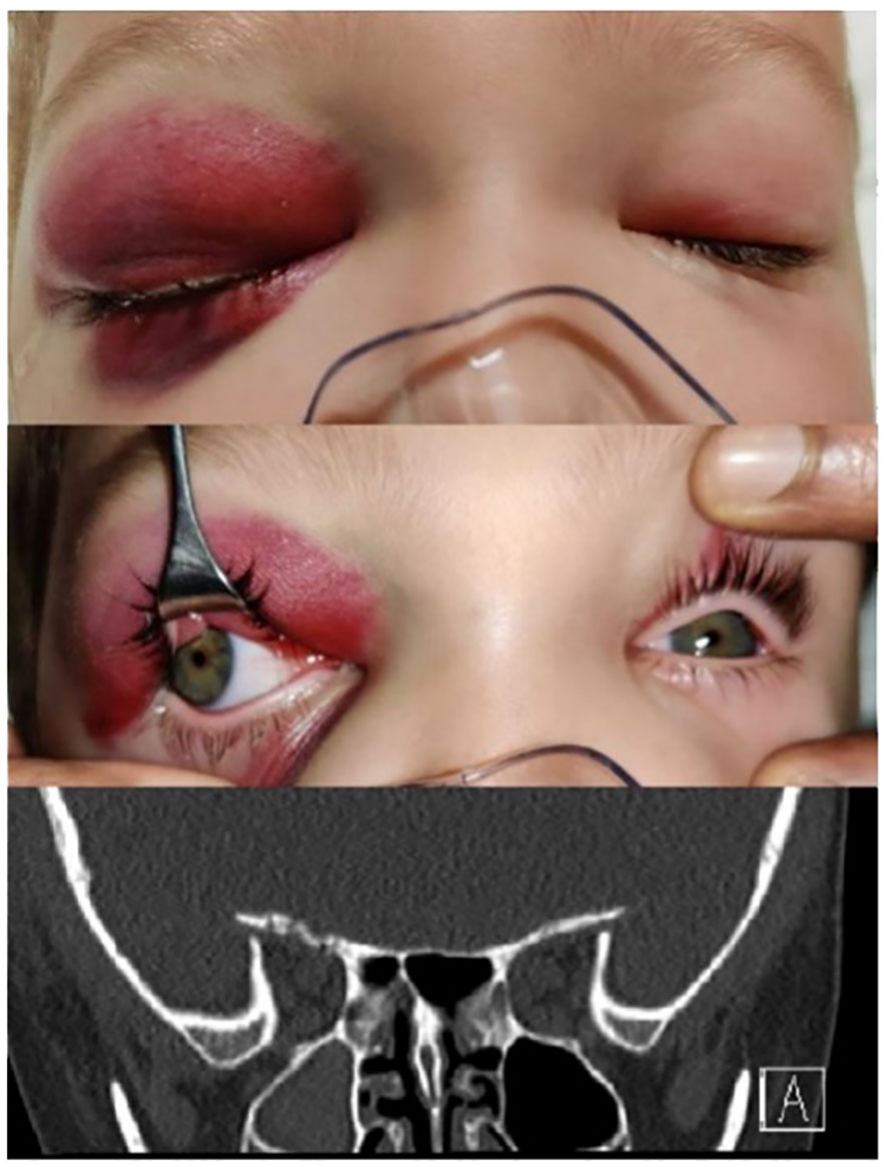
Cranio-orbital fracture of the right side in a 4-yr old child with coronal CT scan of orbital apex showing roof fracture near the superior orbital fissure adjacent to the anterior cranial fossa.

Complex orbital fractures comprised 25.6% (n=11) of the cases. These orbitofacial fractures involved not only the orbital rims with variable degrees of orbital wall involvement but also adjacent bones of the craniofacial skeleton system. These included zygomatico-maxillary complex (ZMC) which often involves the lateral wall and floor (**Figure 7**), naso-orbito-ethmoid (NOE) fractures with involves the medial wall, cranio-orbital fractures (which involves the orbital roof and the anterior cranial fossa (**Figure 8**), and Le Fort type fractures (which involve the orbital floor and medial wall alone (Type II) or the medial and lateral walls (Type III) respectively). These fractures result from high-energy impacts such as RTAs or falls from heights and required early, multidisciplinary and extensive surgical interventions. ZMC fractures were the most common among complex fractures at 45.4% (n=5), followed by cranio-orbital fractures at 27.3% (n=3) and Le Fort type fractures at 27.3% (n=3) equally.

#### 3.1.6 Management outcomes

As in most orbital fractures, patients were managed conservatively whenever possible. However, a significant proportion of pediatric orbital fractures underwent surgical intervention - 65% (n=28) due to clinically significant soft tissue entrapment with diplopia or vasovagal response in 56% (n=24) or enophthalmos. The remaining 35% (n=15), were conservatively managed with expectant recovery. Regarding timing of intervention, 53% (n=23) were performed within 24 hours, 18.6% (n=8) within the week, 21% (n=9) within the month, and 7% (n=3) after a month. For those who underwent surgery (n=28), a bioresorbable implant (Rapidsorb^®^, Osteomesh^®^, Polymax^®^, MacroPore^®^) was used in 64.3% (n=18) of cases, while 25% (n=7) received permanent implants (Titanium meshes and plates, Medpor^®^). Interestingly 10.7% (n=3) received no implants. All patients who underwent surgery showed clinical improvement – both related to diplopia and enophthalmos. No implant or surgery related complications were encountered in our study regardless of the nature of implants. However 3 patients (1.7%) had residual diplopia in extreme upgaze at last follow up, 2 patients had mild residual enophthalmos (2mm difference), and 1 teenager developed depressive mood changes related to the violence related to the orbital fracture.

### 3.2 Inferential statistics

#### 3.2.1 Chi-square test: laterality and outcomes

**Figure 9:**
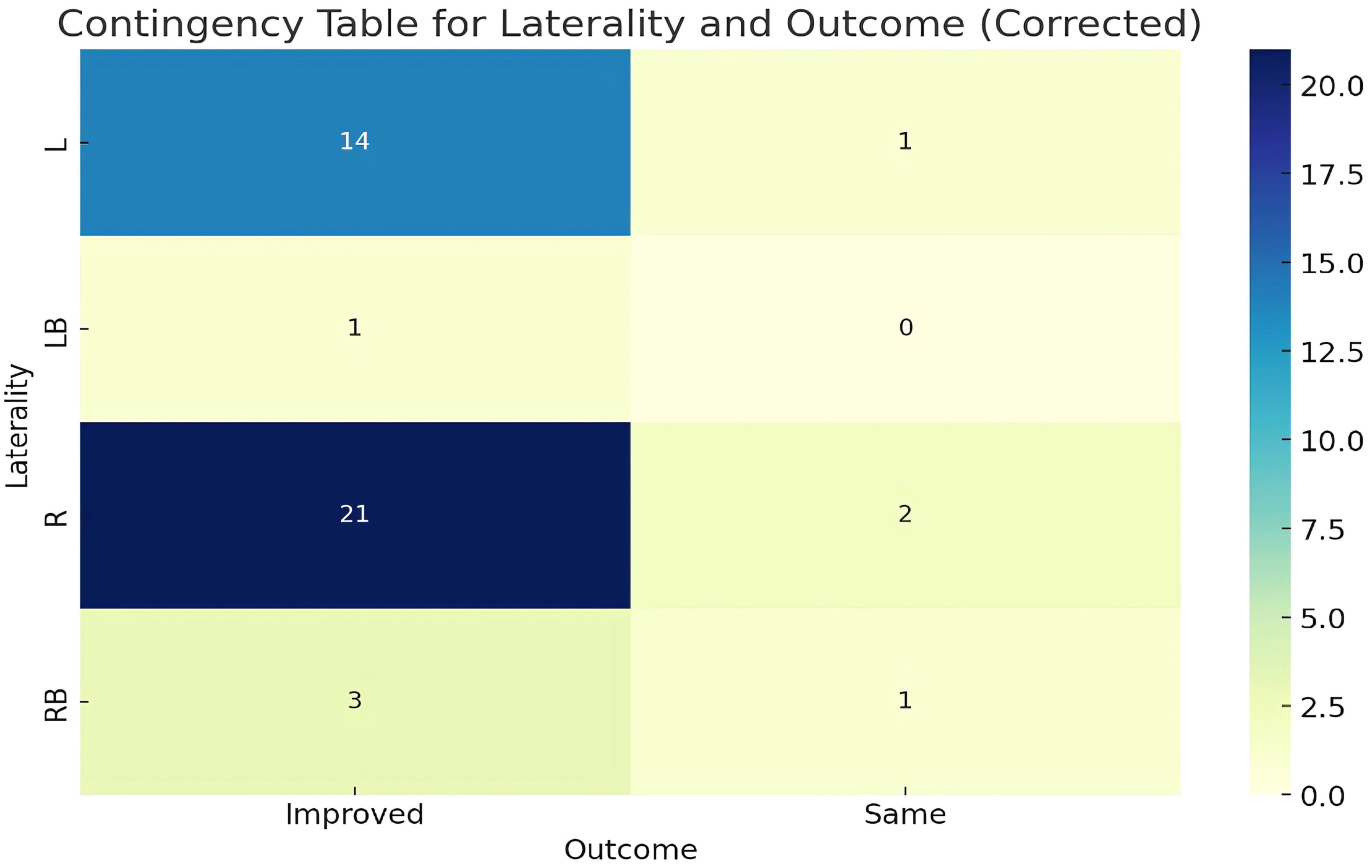
Contingency table for laterality and outcome.

The contingency table shown in **Figure 9** provided an analysis of the relationship between the laterality of fractures (L, LB, R, RB) and the treatment outcomes (Improved, Same). After correcting the inconsistencies in the ‘Outcome’ data, the Chi-square test results indicated a Chi-squared value of 1.4044 with a p-value of 0.7045 and 3 degrees of freedom. The p-value, being significantly higher than the typical threshold of 0.05, suggests that there was no statistically significant association between the laterality of the fracture and the treatment outcome.

#### 3.2.2 T-Test: age comparison between improved and same outcomes

**Figure 10:**
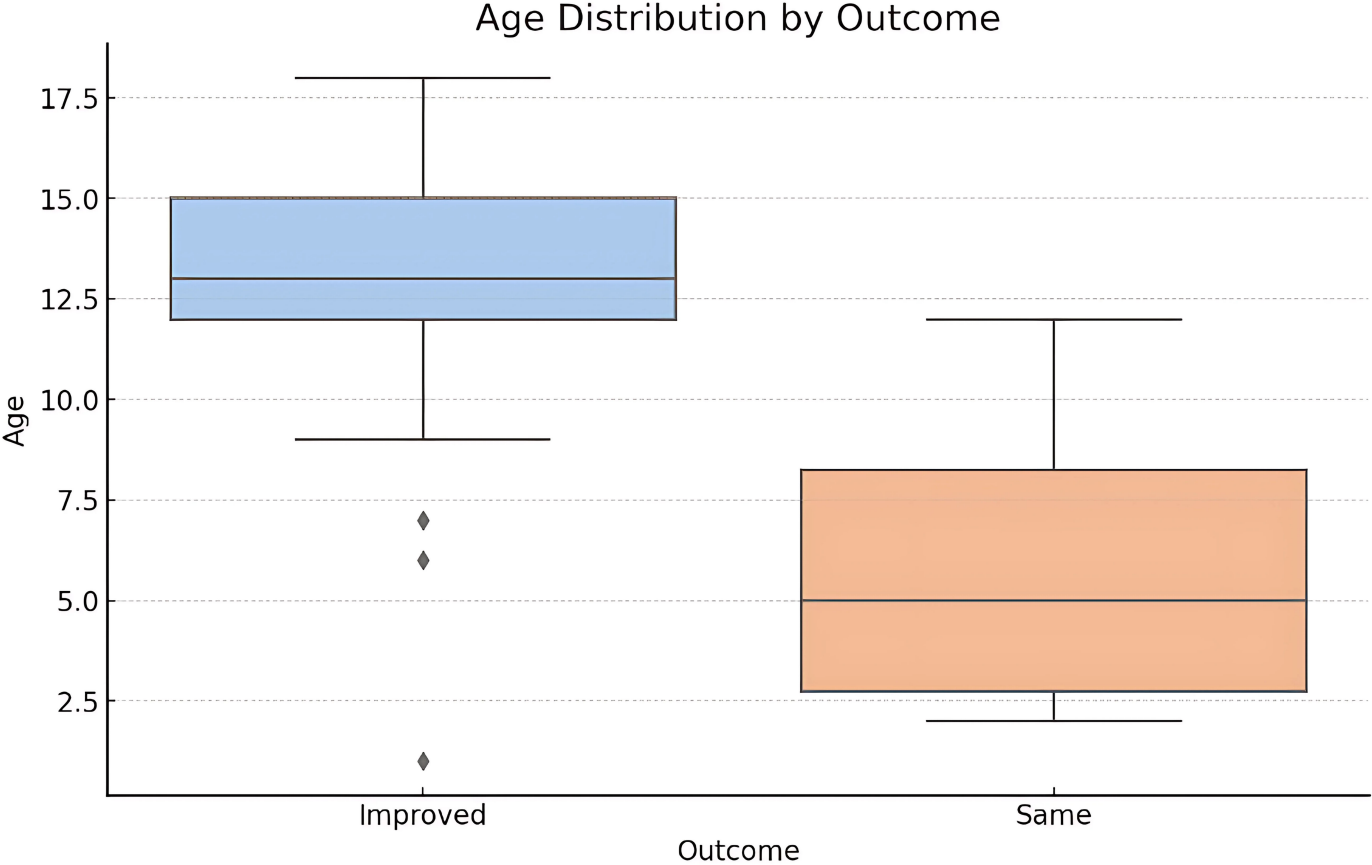
Age distribution by outcome.

The T-Test results show a T-statistic of approximately 3.32 (p-value = 0.002) suggesting significant difference in the mean age between patients who showed complete improvement and those who did not. The boxplot shown in **Figure 10** shows that patients who had an “Improved” outcome tend to be younger compared to those who had not. The median age for improved outcomes is lower, and the interquartile range is also narrower. This visualization supports the finding from the T-Test that age may be a significant factor in treatment outcomes.

#### 3.2.3 ANOVA: age comparison among different etiologies

**Figure 11:**
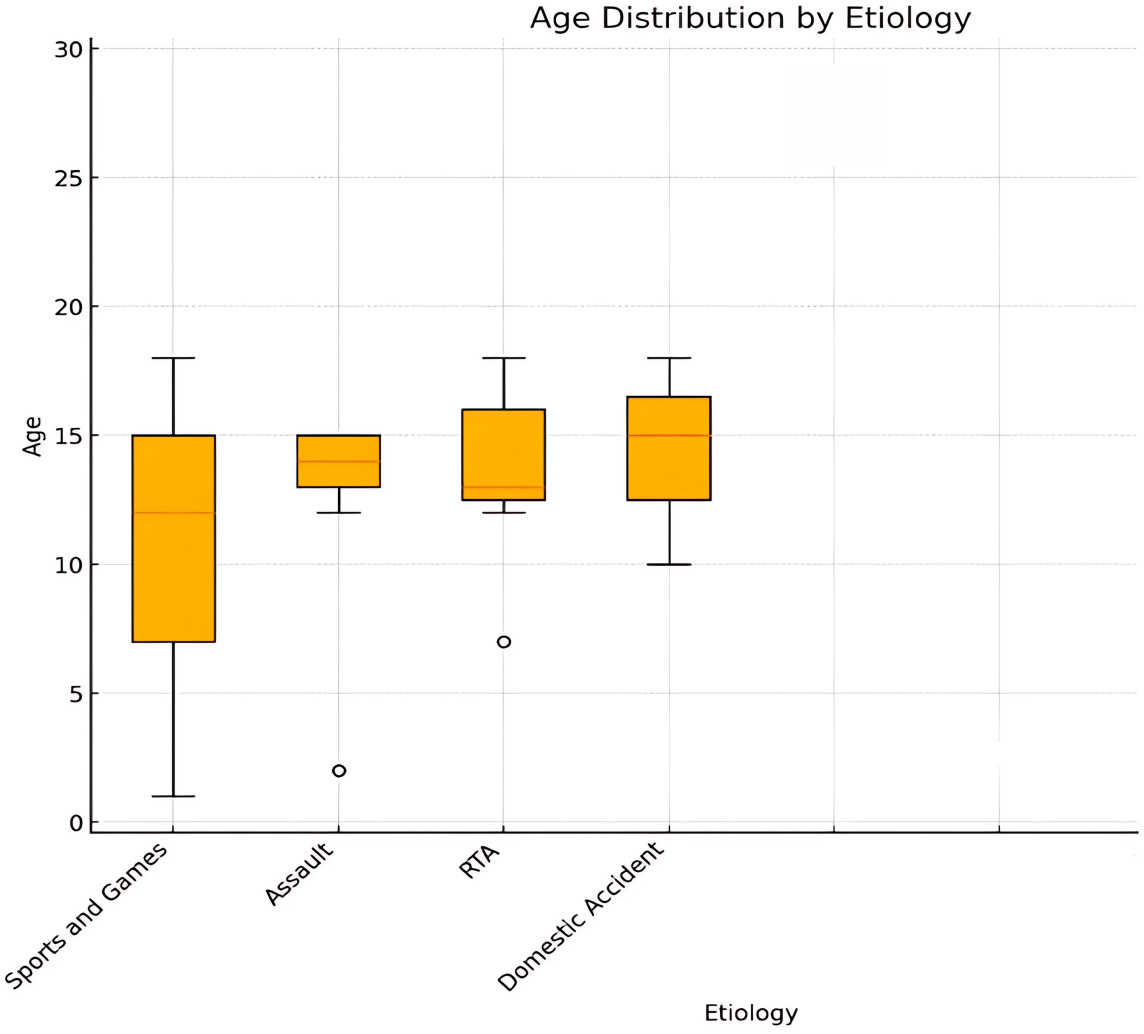
Boxplot of age by etiology.

The ANOVA results show an F-statistic of approximately 5.02 (p-value < 0.001) demonstrating a significant correlation between age and etiologies (**Figure 11**). For example, fractures caused by sports and games tend to occur in older children and adolescents, while those caused by falls are more common in younger children.

**Figure 12:**
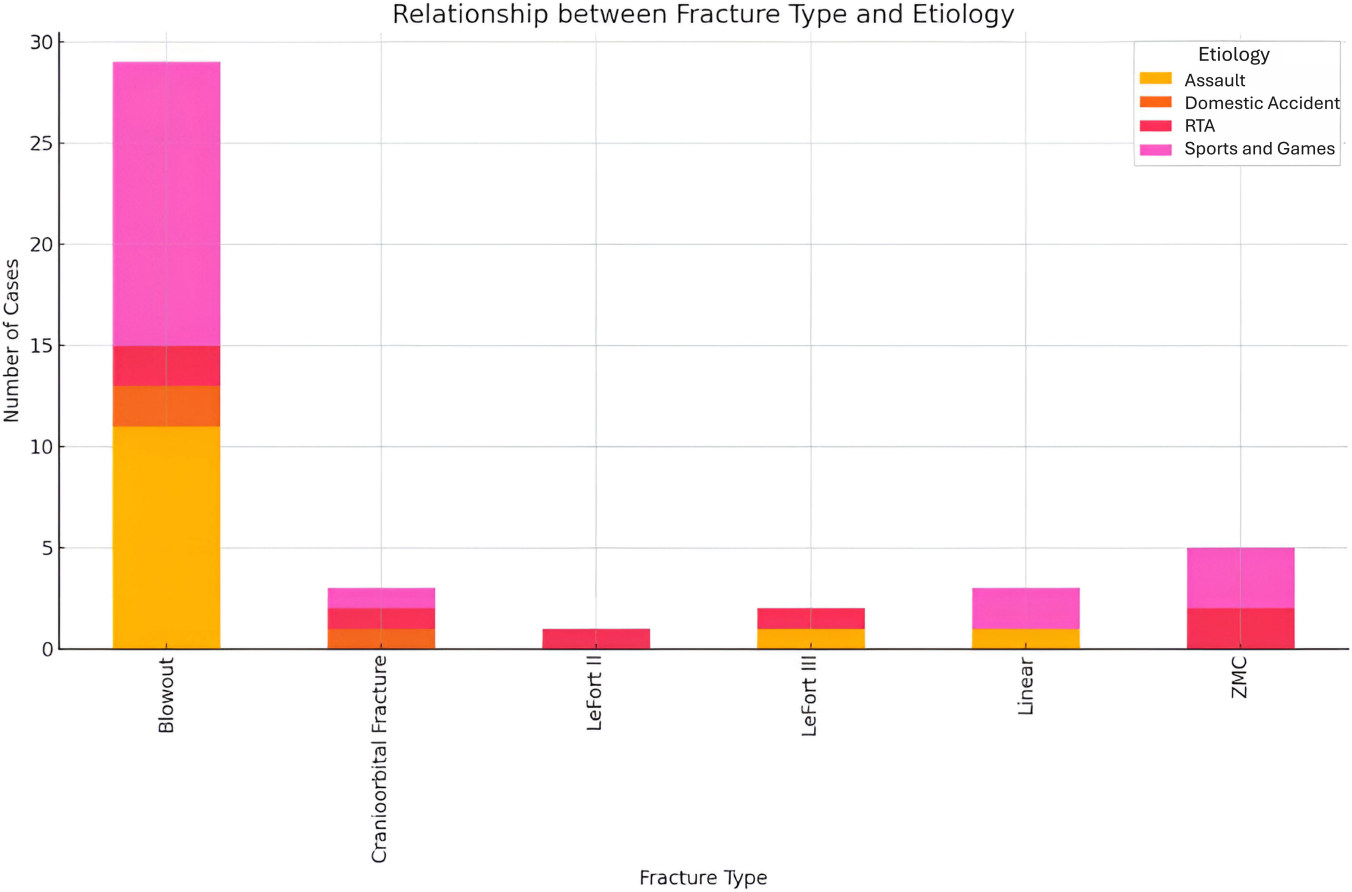
Relationship between fracture type and etiology.

Analysis of the relationship between fracture type and etiology revealed significant insights into the causes of different fracture type (**Figure 12**). Blowout fractures were most frequently associated with sports, games and assault, indicating that high-energy impacts, whether from recreational sports activities or resultant physical altercations. Cranio-orbital fractures were less frequent but were linked to high velocity and impact incidents such as road traffic accidents (RTA) and, to a lesser extent, domestic accidents. ZMC fractures on the other hand were also commonly seen in sports, games and RTAs, indicating that both recreational activities and vehicular accidents contribute significantly to these injuries.

**Figure 13:**
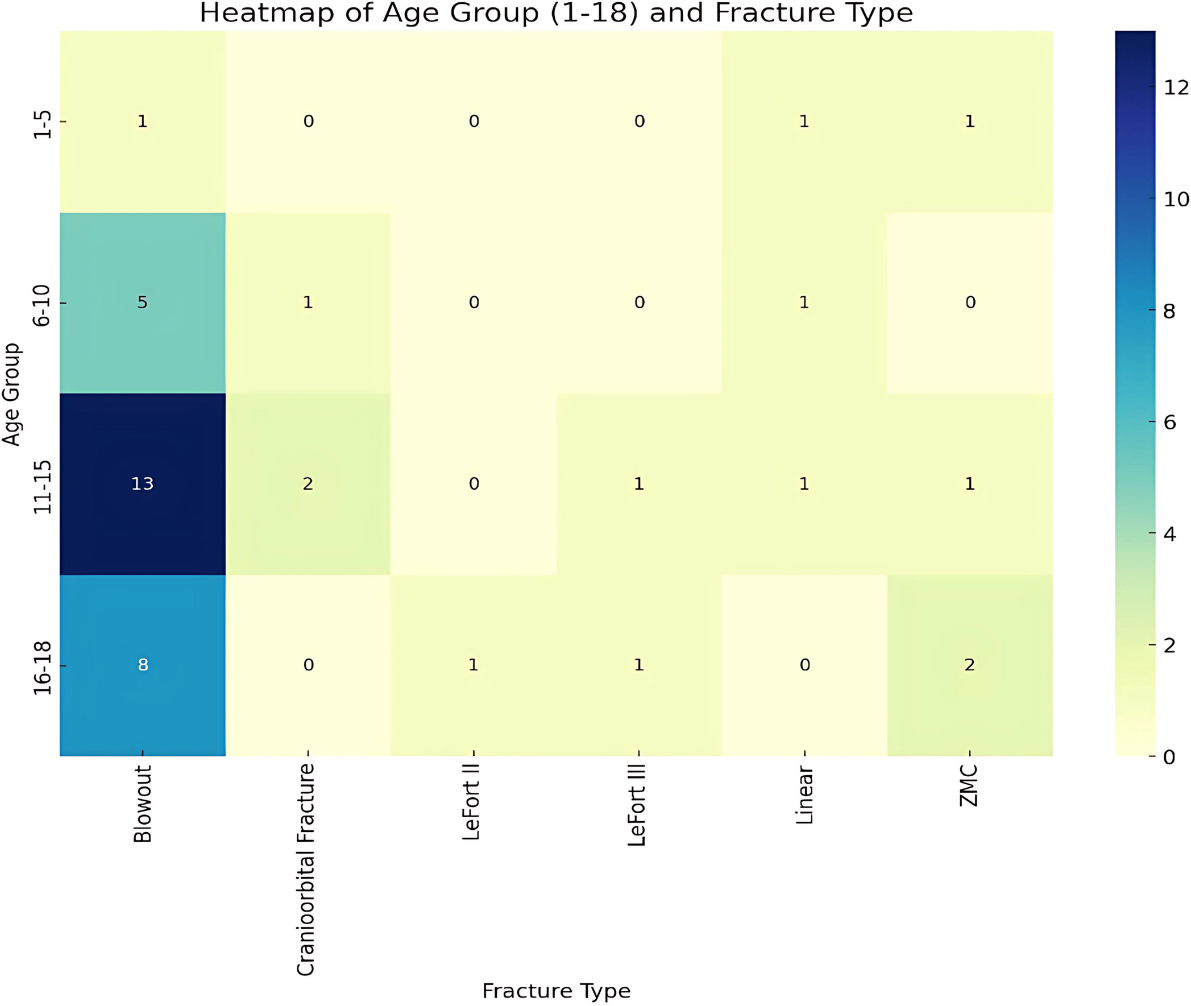
Heatmap of age group and fracture type.

The heat map analysis of pediatric fracture data shown in **Figure 13** was segmented into age groups 1-5, 6-10, 11-15, and 16-18 years, revealing that fracture types vary by age-group. The youngest group (<5 years) had a balanced distribution of fracture types. The remaining age groups showed a predominance of blow-out fractures.

A Chi-Square test indicated no statistically significant relationship between age groups and fracture types (p-value = 0.11376). Despite observable trends, such as the prevalence of blow-out fractures in older children, these were not statistically significant.

## 4 Discussion

Despite pediatric orbital fractures constituting only around 15% of all pediatric fractures (4), the demographics behind these fractures—beyond the orbital floor—are seldom documented in the literature. As in adults, previous studies also report a male predominance in children as well, which is consistent with our study albeit higher percentages than previously reported (8, 15, 16). Regarding predisposing factors, activities of daily living (ADL) were previously reported as the most common cause of pediatric orbital fractures followed by RTAs although they were not stratified by age (8). In our Singapore study, a resource-rich nation where children are generally in a safe environment and majority of them are at school, the most common cause of orbital fractures were games and sports-related activities, especially in the younger age group (< 7 years of age). While consistent with our publication from 2012 (17), this is reportedly different from earlier publications where falls were reported as the most common cause in the younger age group (8). Such disparity in etiology in various nations even within Asia-Pacific probably reflects that children may not be at school and may even be part of the workforce especially in less-regulated nations. Interestingly, assault and violence were the second most common cause in our study, in contrast to a publication from North America where it was the least (8). This observation could be related to greater social interaction, the more competitive nature of sports-related activities and possibly even exposure to social and mainstream media where violence takes centerstage. Road traffic accidents were the third most common cause in our study, as opposed to others earlier where it was found to be more common (8, 15, 16), once again possibly related to road traffic regulations, enforcement and discipline amongst children in the study.

Although majority of the orbital fractures in our study were pure orbital blowout fractures consistent with other publications, our study showed a quarter had more complex orbitofacial fractures (ZMC, NOE, Le Fort and even cranioorbital fractures) highlighting a significant proportion sustained high velocity or high impact injuries requiring more invasive and often multidisciplinary management. As expected, blunt injury from assaults, sports and games were the most common cause of blowout fractures. High impact and high velocity situations like RTAs and high impact sports such as rugby and activities such as trampolines were the most common cause of complex orbitofacial fractures.

When the fracture type and etiology is stratified by age and educational level, preschool children (< 6 yrs) typically experience injuries from play, and more likely to result in simple blowout fractures. Older primary school children (7-12 yrs) on the other hand experience injuries from sports and recreational activities like ball games and cycling, where blowout and cranio-orbital fractures were frequently seen. Lastly, in the secondary school children in the adolescent age group (13-18 yrs), assault was more common followed by sports and games and road traffic accidents. Complex orbitofacial fractures were also more commonly seen in this age group.

Indications and timing of intervention in our study included and depended on the age, entrapment of orbital soft tissues with diplopia, degree of orbit or orbitofacial deformity including enophthalmos as reported in earlier literature (18). Younger children with clinically significant fractures typically underwent surgical intervention within 24 to 48 hours. Urgent attention was also given to patients with orbital tissue and muscle incarceration (EOM-IMS Complex) for best prognosis (10). Older children who were otherwise stable, had co-morbidities or unstable due to systemic polytrauma (neurologic injury, multiple organ involvement, multiple fractures, etc.) or where parents were undecided were delayed, but no longer than 2 weeks. Open reduction with internal fixation of orbital fractures wherever possible through a direct transconjunctival approach was the most common procedure with preference for bioresorbable implants for children with small- medium sized fractures involving one or two walls. Complex orbitofacial fractures often require non-bioresorbable implants especially for the orbital rims (titanium mini plates) and received multidisciplinary care requiring close coordination. As with previous studies (18), our study shows that the earlier the treatment in children, the better was the outcome.

Children who underwent surgery demonstrated both subjective and objective improvements immediately postoperatively and during follow-up (up to five years). Diplopia and pain improved as early as the immediate postoperative period, with the maximum recovery occurring within six weeks. Subjective improvements were observed in all patients, including the resolution of diplopia, nausea, and pain. Objective improvements were reflected with resolution of enophthalmos and repair of associated soft tissue injuries. Patients with blowout fractures generally had favorable outcomes, consistent with findings from previous studies (19).

**Figure 14:**
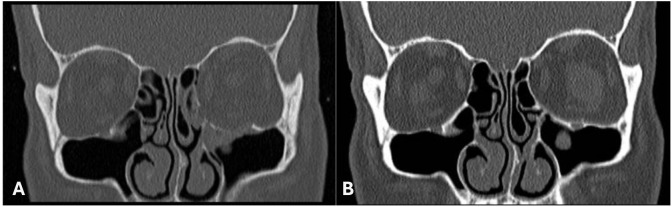
**(A)** Pre Operative Right Orbital Floor Fracture. **(B)** 18 months Post Repair of Right Orbital Floor Fracture with a Bioresorbable Implant.

To those who underwent surgery with implants, the study found no difference in clinical improvement between bioresorbable and permanent implants. Bioresorbable implants used include Rapidsorb^®^, Osteomesh^®^, Polymax^®^, and Macropore^®^ while permanent implants included Medpor^®^ and Titanium implants. No cases of infection, palpability or extrusion were seen with bioresorbable implants, consistent with existing literature (20). Furthermore, new bone growth was seen in most patients with bioresorbable implant on late follow up imaging (15-18 months), with restoration of orbital shape, contour and symmetry (**Figure 14**). Given these findings, we advocate the use of bioresorbable implants for most pediatric orbital fractures wherever available. Some of these bioresorbable implants are thermolabile offering malleability, allowing 3-dimensional contouring much like a patient specific implant. Permanent implants still serve an important role in more complex large multiple wall fractures with a high likelihood of repetitive trauma such as in contact sports.

The study has several limitations inherent to its retrospective design and the rarity of pediatric orbital fractures. The reliance on existing records introduces the potential for incomplete documentation, thereby restricting the variables available for analysis. Additionally, as a single-institution study conducted in Singapore, where the population is predominantly of Chinese ethnicity, the findings may be subject to selection and information biases, potentially limiting their generalizability to more diverse populations.

## 5 Conclusion

Although simple pediatric orbital blowout fractures are still the most common among pediatric patients, the study showed that a quarter of them can still present with complex orbit and orbitofacial fractures. Most of these pediatric orbital fractures are seen in males and associated with age (secondary school) and increasing play and activity. The study also showed that early intervention and when required a multidisciplinary approach is crucial to good outcomes. The study also underscores the increasing role of bioresorbable implants in reconstruction of the pediatric orbital fractures, with reduced implant-related complications.

This study hopes to provides critical insights to improve clinical outcomes in pediatric orbital fractures, optimize healthcare resources, and guide trauma prevention strategies. Understanding its demographics and etiology can refine management protocols, improve patient care and surgical decision-making. Identifying the etiology, such as play injuries or falls, informs targeted public health initiatives and safety regulations. Regional epidemiological data facilitate comparisons with national and global trends, highlighting unique risk factors and informing policy development. Ultimately, this research contributes to evidence-based policies, injury prevention programs, and improved long-term outcomes for pediatric orbital fracture patients.

## Data Availability

The raw data supporting the conclusions of this article will be made available by the authors, without undue reservation.
